# Use of high-flow nasal canulae: effect on alveolar pressure and its limitation

**DOI:** 10.1186/cc12029

**Published:** 2013-03-19

**Authors:** H Hayami, K Mizutani, M Shioda, S Takaki, H Maejima, K Ueno, Y Yamaguchi, T Kariya, T Gotoh

**Affiliations:** 1 Yokohama City University Hospital, Yokohama, Japan

## Introduction

High-flow nasal canulae (HFNC) deliver high-flow humidified gas at up to 60 l/minute. There are two types of respiratory circuit to generate mix gas flow, Blender type (typeB) and Venturi type (typeV). The therapy is well established in the pediatric population and HFNC use has been described in the adult population. It has been reported that HFNC provide higher FIO_2 _compared with low-flow canulae, and also create mild positive pharyngeal airway pressure, but the effect on alveolar pressure is unknown. We aimed to investigate the effect of HFNC on alveolar pressure, by measuring intratracheal pressure in patients with a cricothyrotomy catheter (CTC). At the same time, we measured the actual gas flow rate (AGFR) by flowmeter and compared it with assumed flow.

## Methods

Seven patients with a CTC were participated. A tube was connected to the CTC and the tube was then connected to a pressure transducer to measure intratracheal pressure. The HFNC (Optiflow system) were applied with the humidiffer to optimize humidication. TypeB was used in three patients and typeV in four patients. The flow was started at 10 l/minute. This flow rate was titrated upwards to a maximum of 60 l/minute (10, 25, 30, 40, 50, 60 l/minute) and the AGFR was measured. Intratracheal pressure tracing was done over 1 minute. Airway pressure measurement was repeated and the maximal expiratory pressure was measured in mmHg.

## Results

The AGFR in the respiratory circuit was almost same in typeB, but there was obvious decrease in the AGFR in typeV (7.1 ± 1.0, 17.7 ± 0.8, 21.9 ± 0.9, 29.9 ± 3.6, 36.9 ± 2.7, 45.0 ± 5.5 l/minute at assumed flow, 10, 25, 30, 40, 50, 60 l/minute, respectively). HFNC significantly increased maximal expiratory pressure in both groups, 1.5 ± 2.1, 2.0 ± 1.0, 3.0 ± 2.8, 4.5 ± 3.5 mmHg for typeV and 2.5 ± 0.7, 5.8 ± 2.4, 6.0 ± 2.8, 8.0 ± 2.8 mmHg (maximum 10 mmHg) for typeB, when AGFR was set at 30, 40, 50, 60 l/minute. Higher AGFRs were found to result in larger increase in maximum expiratory pressure. The data indicate that HFNC are associated with an increase in intratracheal expiratory pressure. Because it was difficult to determine end-expiratory pressure, we chose maximal expiratory pressure for a substitute. The reason why AGFR in typeV was lower than assumed flow may be the resistance generated by NC. The larger increase in expiratory pressure in our study than previously reported may be due to the effect of high respiratory resistance of Japanese who have relatively small airway structure compared with western people.

## Conclusion

HFNC are effective in providing higher expiratory pressure. It is important to know the flow rate is lower than expected when the Venturi type is used.

